# Response to: Alpha Rhythm, Alpha Coma and Entropy in EEG

**DOI:** 10.1097/WNP.0000000000001178

**Published:** 2025-06-17

**Authors:** Giulio Degano, Pia De Stefano

**Affiliations:** Department of Intensive Care, Neuro-Intensive Care Unit, University Hospital of Geneva, Geneva, Switzerland.

***In Reply:***

We appreciate the opportunity to respond to Velazquez et al.'s thoughtful comments. Although we agree with their main points about using the entropy and complexity metric for EEG analysis, we wanted to explain further the choices made for the study.^1^

In Velazquez et al., the authors highlight that our specific pipeline of analysis was prepared for a specific use case, and this choice has impacted the estimate of the sample entropy measure. Although we agree with this comment, the reason behind these choices was to focus on the alpha rhythm per se and both in terms of power^2,3^ and entropy.^4,5^ First, the bandpass filtering and, importantly, the resampling factor of the EEG were scaled to an appropriate and predefined range to better track alpha fluctuation. This was an essential step in the analysis because the study's goal was not to simply investigate the sample entropy across multiple scales, as done in previous work on coma states,^6,7^ but the entropy related to alpha oscillation itself. Second, the selection of the region of interest was again used to assess a fair comparison between the two cohorts. Indeed, because alpha-coma state is often characterized by an anteriorization of the alpha network, we decided to select the EEG channels that presented the strongest alpha activity for the pathologic and healthy cohort in a complete data-driven way. We believe that this agnostic approach allowed for a better understanding of the underling differences across the two different entropy levels. It is worth noting that the result remained significant using also different regions (see **Supplementary materials, Supplemental Digital Content 1**, https://links.lww.com/JCNP/A306).

Finally, we chose to compare alpha-coma activity with the eyes-closed condition in healthy subjects primarily because our goal was to investigate entropy within the alpha rhythm as an oscillatory pattern readily identifiable through standard visual raw EEG analysis. There are three clinical contexts in which alpha rhythm is typically appreciated via standard EEG inspection: alpha-coma, posterior background alpha in awake eyes-closed individuals, and drug-induced alpha activity.

As emphasized by Velazquez et al., entropy levels differ significantly between eyes-open and eyes-closed states in healthy individuals. This variation reflects the phenomenon of “EEG reactivity,” wherein posterior alpha activity diminishes or disappears upon eye opening.^2,3^ However, including an eyes-open condition as a control was neither feasible nor appropriate for our study. Our patients were unable to open their eyes or receive visual stimulation, and since our interest lay in entropy associated with visible alpha rhythms at raw EEG, the eyes-open condition would have introduced a substantial confounding factor.

We, therefore, compared alpha-coma with the posterior background alpha observed in awake eyes-closed individuals. Despite differences in network spatialization,^8–10^ both rhythms appear quite similar under standard clinical EEG analysis. In both cases, the alpha activity is spontaneous, and in the alpha-coma condition, the patients also had their eyes closed, further supporting the rationale for this comparison.

It is also unlikely that adding an additional cohort of subjects under different levels of anesthesia would have changed the results, because the alpha rhythms in such cases would no longer be spontaneous but drug-induced. Moreover, drug-induced alpha activity has been repeatedly shown to be more predictable than that observed in healthy, awake individuals.^11–13^

We agree with Velazquez et al. that these steps inevitably affect the assessment of the variability of an EEG signal (as also shown by their simulations), but because the choices of analysis were statistically orthogonal to the two cohorts, we believe that the study's implications are still valid.

Like Velazquez et al., we also believe that this difference in variability across the two cohorts is intriguing, but our interpretation differs slightly from what is suggested by Velazquez et al. We indeed interpret the higher level of entropy in alpha-coma as an underlying generator of cortical activity that is, by its nature, unpredictable. In other words, the lower entropy observed during eyes-closed conditions in healthy participants should not be interpreted as a deeper shutdown compared with alpha-coma. Instead, the variability of the alpha rhythm in alpha-coma states likely reflects an epiphenomenon of an autonomous and erratic rhythmic activity, which is inherently more variable because of its pathologic origin.

## Supplementary Material

SUPPLEMENTARY MATERIAL
